# Comparing sequences without using alignments: application to HIV/SIV subtyping

**DOI:** 10.1186/1471-2105-8-1

**Published:** 2007-01-02

**Authors:** Gilles Didier, Laurent Debomy, Maude Pupin, Ming Zhang, Alexander Grossmann, Claudine Devauchelle, Ivan Laprevotte

**Affiliations:** 1 Institut Mathématique de Luminy, UMR 6206, Campus de Luminy, Case 907, 13288 Marseille Cedex 9, France; 2 Equipe Bioinfo, LIFL, USTL, cité scientifique, Batiment M3, 59655 Villeneuve d'Ascq, France; 3 Department of Bioinformatics, Institute of Microbiology and Genetics, University of Goettingen. Goettingen 37077, Germany; 4 Theoretical Biology and Biophysics Group, Theoretical Division, Los Alamos National Laboratory, Los Alamos, NM, 87545, USA; 5 Laboratoire Statistique et Génome, UMR 8071, Tour Evry 2, 523 Place des Terrasses, 91034 Evry, France

## Abstract

**Background:**

In general, the construction of trees is based on sequence alignments. This procedure, however, leads to loss of informationwhen parts of sequence alignments (for instance ambiguous regions) are deleted before tree building. To overcome this difficulty, one of us previously introduced a new and rapid algorithm that calculates dissimilarity matrices between sequences without preliminary alignment.

**Results:**

In this paper, HIV (Human Immunodeficiency Virus) and SIV (Simian Immunodeficiency Virus) sequence data are used to evaluate this method. The program produces tree topologies that are identical to those obtained by a combination of standard methods detailed in the HIV Sequence Compendium. Manual alignment editing is not necessary at any stage. Furthermore, only one user-specified parameter is needed for constructing trees.

**Conclusion:**

The extensive tests on HIV/SIV subtyping showed that the virus classifications produced by our method are in good agreement with our best taxonomic knowledge, even in non-coding LTR (Long Terminal Repeat) regions that are not tractable by regular alignment methods due to frequent duplications/insertions/deletions. Our method, however, is not limited to the HIV/SIV subtyping. It provides an alternative tree construction without a time-consuming aligning procedure.

## Background

Almost all existing methods for comparing biological sequences are based on certain implicit (and necessary) assumptions about the kinds of changes sequences undergo during their putative evolution. Under these assumptions, some changes like permutations and inversions are often ignored (see however [1-3]) while other kinds of changes (insertions/deletions, for instance) are poorly evaluated because they do not follow regular evolutionary models. As a result, the regions in which these changes occur are often treated as ambiguous and are deleted from sequence alignments before further evolutionary analysis. In addition, as no single alignment method copes with tremendous variety of sequence data, expert editing (manual alignment) becomes often necessary. This is particularly important if the sequences have successive duplications/deletions [4,5], and permutations.

To solve these problems, there has been work on methods of comparing sequences without alignments. An intuitive way of comparing sequences without alignments is to compare their compositions, i.e. the frequency of nucleotides or amino acids appearing in the sequences, as two sequences with significantly different compositions cannot be closely related. However, it is also true that two unrelated sequences can have very similar compositions. So the next step is to consider all *N-words *in a set of sequences (an *N-word *consists of *N *consecutive letters in a sequence) to calculate sequence dissimilarities [6]. This can show some evolutionary relationships. The problem is that the only straightforward relationship between two words is that they are either identical or different. This can be compensated by considering «imperfectly matched words». The problem is then a combinatorial explosion: as we all know, there are too many ways of being imperfect.

To track the evolutionary information embedded in ambiguous regions of sequence alignments and to alleviate the burden of manual editing, one of us developed a method that allows comparison by considering «local decoding of order *N*» of sequences (also called «*N*-local decoding method»), and keeping information of each set of overlapping (step 1) *N*-words that cover each site in a sequence [7]. The method is recapitulated in a recent work describing a new algorithm that computes the «local decoding of order *N*» of a sequence (or a set of sequences) with a complexity linear in sequence length, both in time and memory space [8].

The method was found useful for assessing a manually constructed HIV (Human Immunodeficiency Virus) LTR (long terminal repeat) multiple alignment, which is homology blocks based [5]. «Related sites» were found to correspond to homology blocks between compared sequences, leading us to think that the comparison of the sequences «composition» in «locally decoded letters» could yield a new method for calculating dissimilarities between sequences without an alignment and consequently without taking into account the order of homology blocks in each sequence.

The new method has also been tested against 10 reference alignment-based dissimilarity matrices calculated from 10 multiple alignments of DNA or RNA sequences: it gives alignment-free dissimilarities in very good agreement with the reference alignment-based dissimilarity matrices (better than the correlation obtained when the alignment-free dissimilarity matrix was calculated from the comparison of sequence compositions in words of length *N*) [8].

The aims of this paper are: (A) to give a concise description of this new method (see the section **Methods**) (B) to apply it to a problem of biological interest, namely the classification of HIV/SIV, two of the most variable organisms known so far (see **Results **and **Discussion**).

## Results

### The *N*-local decoding method on complete genome sequences – tests on HIV/SIV complete sequences

**Figure 1:**
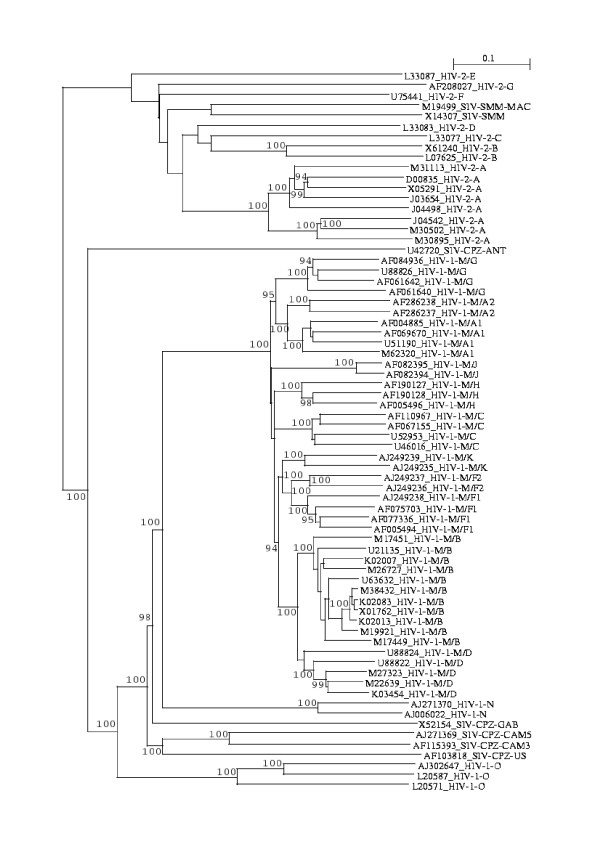
**The neighbor-joining tree obtained from 70 HIV/SIV nucleotide sequences (distance matrix calculated by using the *N*-localdecoding method for *N *= 15)**. The sequences names are written as follows: their GenBank accession numbers, followed by their nomenclature names [9–11, 14, 15]. These sequences can be retrieved from the Los Alamos HIV sequence database [24]. Bootstrap values (≥ 90%) are indicated.

The *N*-local decoding method was used to calculate dissimilarity matrices for all 70 sequences described in Methods section. Four incomplete genome sequences (HIV-2 subtype C, D, E, F) were also included here because these sequences may have kept strong subtyping signals that are in the complete genome sequences. Figure 1 shows a tree obtained from the dissimilarity matrices calculated by our method. Two types of HIV, representing independent cross-species transmission events [9], are clearly distinguished: HIV-1 is closer to SIV-CPZ, and HIV-2 is closer to SIV-SMM. Nine subtypes of HIV-1 group M cluster distinctly, with sub-subtypes significantly more closely related to each other (A1 and A2, F1 and F2, B and D – though B and D are regarded as subtypes instead of sub-subtypes for historical reasons [10]). Subtype K is more distant from sub-subtypes F1 and F2 than these are from each other, but closer to F1 and F2 that to other subtypes, i.e., in the range of subtype B and D distances [11]. HIV-1 groups N and O are in the expected distances from the M group. The N group intercalates between HIV-1 group M and SIV-CPZ (CPZ-CAM3, -CAM5, -GAB and -US), consistent with a suggestion that group N is a recombinant between a SIV-CPZ strain and a virus related to the ancestor of group M [12]. The group O is intercalated between these CPZ (CPZ-CAM3, -CAM5, -GAB and -US) and CPZ-ANT that is the borderline in the HIV-1/SIV-CPZ lineages [13]. HIV-2 subtypes also form clear clusters, respectively, including subtypes C, D, E, and F that are about half of *gag *region.

Consequently, the topology of the tree shown in Figure 1 agrees very well with existing knowledge [9-11,14,15] and recognizes significant HIV/SIV evolutionary events.

The computational program used to calculate the dissimilarity matrices from which the trees can be constructed has been tested for a wide range of values of *N*, which is the only user-specified parameter of the local decoding method. When *N *ranges from 13 to 35, there is no significant change in the tree. Furthermore, the topology of the tree obtained by *N*-local decoding (*N *= 13–29) of 46 HIV/SIV complete nucleotide sequences, is identical with the complete genome tree available from HIV/SIV sequence compendium 2000 (compare Figure "Comp2000_13_tree" corresponding to the file "Comp_2000" at [16] with the published tree at [17].

### The *N*-local decoding method on short sequences – tests on HIV/SIV *gag*, *pol*, *env *and *nef *sequences

HIV/SIV exhibit great variety in different parts of their genomes, with *env *representing the most variable region. All 70 sequences described in the Methods section were used to test different sequence regions. For *gag*, 66 HIV/SIV sequences (only the *gag *region is used) and 4 HIV-2 sequences that cover partial *gag *(sequence length 771–781 nt, in contrast to 1473–1569 nt of the complete *gag *sequences) were tested. For other genome regions, *pol *(2993–3360 nt),*env *(2499–2658 nt) and *nef *(292–783 nt), only 66 sequences were selected because of unavailability of sequences in these regions from HIV-2 subtype C, D, E, F. The trees of these four regions, based on the *N*-local decoding calculated sequence distance matrices (see figures «gag18_tree», «pol22_tree», «env15_tree, and «nef13_tree» at [16]), also agree well with the established HIV/SIV phylogenetic trees in these regions [9-11,14,15] with the exception of *nef*. A few discrepancies exist in *nef*: in our method, HIV-1 group M sub-subtypes F1 and F2 mix together. Subtype K, depending on the chosen value of *N*, either is still close to F1/F2 as expected, or is isolated or loosely related to subtype J. SIV-CPZ-ANT is intermediary between HIV-1-M-N/SIV-CPZ-HIV-1-O and HIV-2/SIV-SMM. These discrepancies may simply reflect uneven sequence complexity in different genome regions, or just reflect the differences in treating ambiguous alignment regions: the *N*-local decoding method keeps all these regions while the traditional alignment-based methods have to delete those alignment regions in order to produce an unbiased tree.

The best orders *N *tested in these regions are: *gag*, *N *= 11 to 23; *pol*, *N *= 11 to 30; *env*, *N *= 12 to 24; and *nef*, *N *= 11 to 20.

### The *N*-local decoding method on sequences that traditional alignment-based methods cannot deal with – tests on non-coding LTR sequences

Forty-three of the 70 sequences (retrieved from the HIV sequence database as described in Methods section) cover the non-coding part of LTR (complete non-coding LTR region or at least the 5' portion of this segment including the polyadenylation signal AATAAA). The length of this part ranges from 211 to 328 nt in the HIV-1/SIV-CPZ subset, and 433 to 508 nt in the HIV-2/SIV-SMM subset. These short non-coding LTR segments contain many duplications/insertions/deletions that make them difficult for traditional alignment-based phylogenic studies [5].

**Figure 2:**
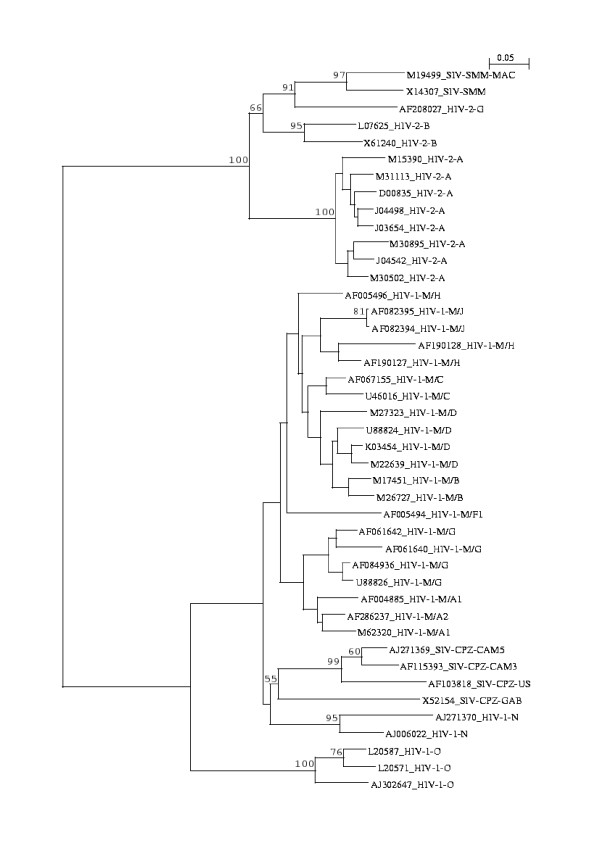
**The neighbor-joining tree obtained from 43 HIV/SIV non-coding parts of LTR nucleotide sequences (distance matrix calculated for *N *= 11)**. M15390 corresponds to the HIV-2-A ROD isolate just as X05291 for Figure 1. Sequence names follow the same rule as in Figure 1. Bootstrap values (≥ 50%) are indicated.

**Figure 3:**
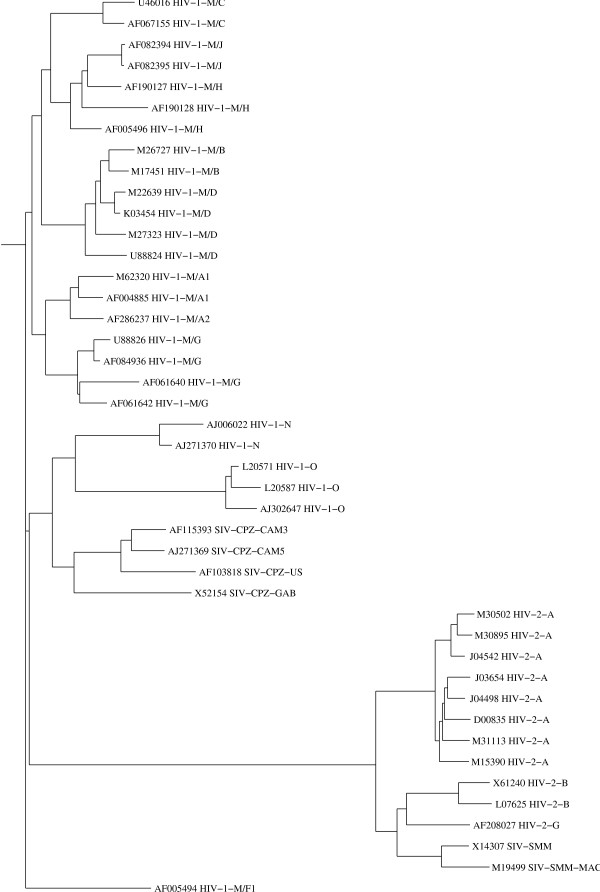
**The neighbor-joining tree calculated from the multiple alignment of the same 43 sequences as in figure 2, produced by the CLUSTAL-W program**. Sequence names follow the same rule as in Figure 1.

**Figure 4:**
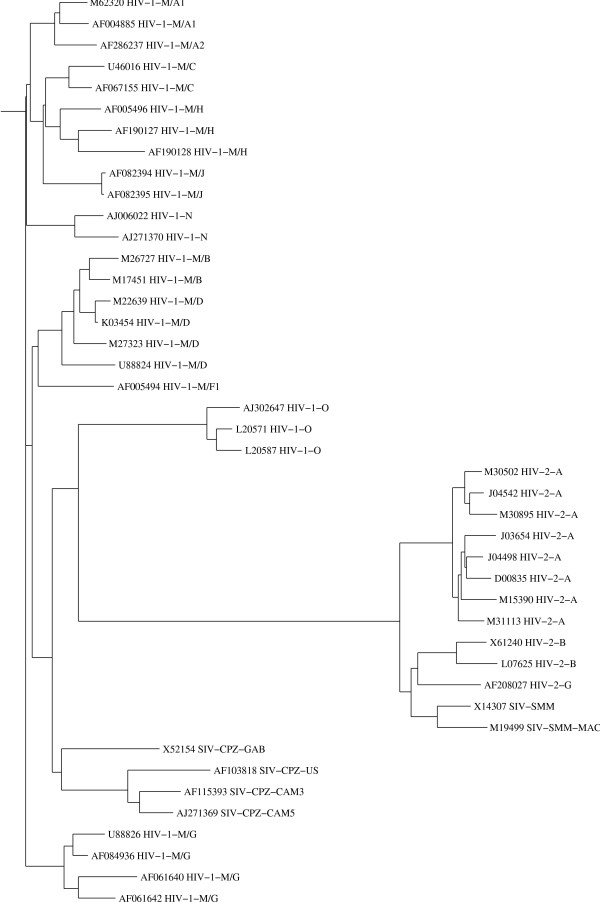
**The neighbor-joining tree calculated from the multiple alignment of the same 43 sequences as in Figure 2, produced by the DIALIGN-2 program**. Sequence names follow the same rule as in Figure 1.

There is no suitable reference tree available for this non-coding LTR region. Thus we built trees based on CLUSTAL-W [18] and DIALIGN-2 [19] alignments and compared them with the *N*-local-decoding-method-based tree. In our method (Figure 2), two types of HIV are very well characterized in terms of defining their relations to their SIV origin (HIV-2/SIV-SMM and HIV-1/SIV-CPZ). The sequence relations among different subtypes of HIV-1, HIV-2, and SIV are clearly defined except for A1 and A2 that are not distinguishable from each other. CLUSTAL-W-based tree (Fig. 3), however, incorrectly clusters HIV-1/SIV-CPZ, and HIV-1 M, N, O groups. In contrast, the tree based on DIALIGN-2 (Fig. 4), a multiple alignment program based on homology blocks, features better HIV clades than CLUSTAL-W-based tree. Subtypes in HIV-1 and HIV-2 are well separated from each other. But this DIALIGN-based tree is far less satisfactory than the N-local-decoding-method-based tree (Fig. 2). For instance, sub-subtypes in HIV-1 group M mix together; HIV-1 group N is located inside HIV-1 group M and it is loosely related to SIV-CPZ; subtype A and G of HIV-1 group M are not closely related to each other as expected. In other words, neither CLUSTAL-W- nor DIALIGN-based tree represent better HIV/SIV sequence relations and distances than the tree obtained by our method.

In our method, bootstrap values strongly support the clustering of HIV-2 (and their subtypes), SIV-SMM, HIV-1-M, HIV-1-N, SIV-CPZ, and HIV-1-O. However, in contrast to Figure 1, the values are low within HIV-1-M. This is not surprising since these sequences are short and very similar to each other. The parameter *N *tested here ranges from 10 to 21 in order to generate the most appropriate neighbor-joining trees for those 43 sequences. For various subtypes, however, this *N *sometimes falls into different ranges. For example, *N *= 9–14, 27–43 and even further make HIV-1 subtype C cluster clearly defined; *N *= 10–60 and beyond are needed for HIV-1 subtype G cluster; *N *= 8–60 and above are necessary to cluster two J sequences of HIV-1-group M. Finally, *N *= 9–17 are needed to clearly cluster HIV-1-M/B and D subtype, and *N *= 19–25 are necessary to distinguish HIV-1 subtype B and D.

In short, our HIV/SIV subtyping results confirmed the good performance of the *N*-local-decoding method, regardless the complexity of genome regions. The *N *values listed above not only are useful in making an *N*-parameter reference for future *N*-local-decoding method applications, but also imply different evolutionary pressures in various HIV/SIV clades.

### Similarity blocks displayed by the *N*-local decoding method – an example of the HIV non-coding LTR sequences

In our previous study of HIV LTR [5], a sequence alignment had to be manually constructed in order to take into account the frequent duplications/insertions/deletions events. This strategy is similar to the *N*-local-decoding procedure described in this paper.

Here we use the HIV non-coding-LTR NF*K*B binding site (GGGACTTTCCA/G) (see [5]) and its flanking sequences obtained from 43 sequences (the same sequences used in non-coding HIV LTR Results section just above) as an example to show the relationship between the similarity blocks and the *N*-classes.

**Figure 5:**
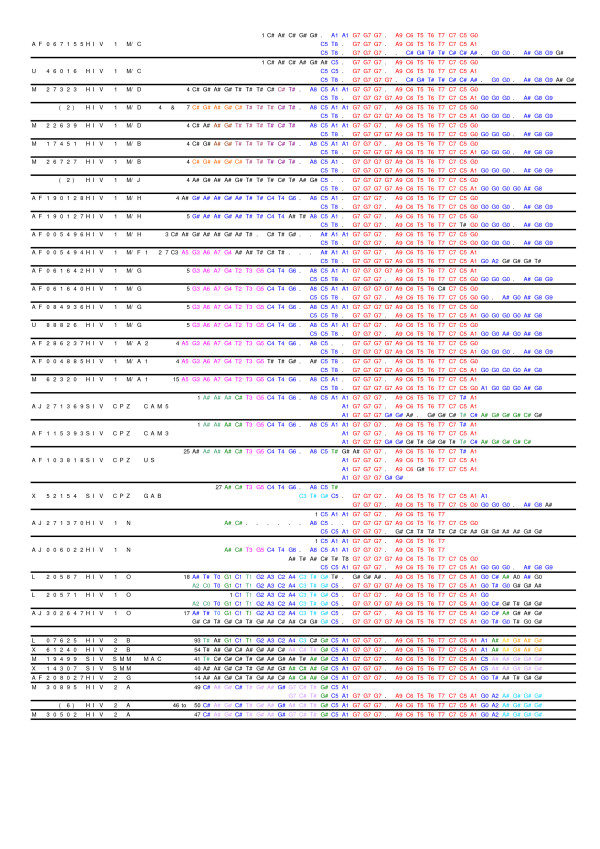
**Similarity blocks found by applying the *N*-local decoding method (for *N *= 11) to the HIV non-coding LTR sequences**. This is a nucleotide sequences alignment of the 43 non-coding LTR sequences that are used in the Results section corresponding to Figures 2-4. The alignment is focused on the NF*K*B binding site (GGGACTTTCCA/G) and its flanking sequences. Most often, the similarity blocks are aligned. In the left columns, the sequences are given by their database accession numbers followed by their nomenclature (HIV-1; HIV-2; SIV-CPZ-CAM 3; -CAM 5; -GAB; -US; SIV-SMM; SIV-SMM-MAC) and their groups, subtypes and sub-subtypes (for HIV-1 and HIV-2). The sequences are re-grouped according to the phylogeny as seen in Figures 1-4. The letters are re-written by applying the *N*-local decoding method (*N *= 11). When the identifier contains a number with more than one digit, this number is replaced by a #. Identical recoded letters that are in the same column are written using the same colour. The sequences are written from the left to the right. The coordinate of the first letter in the file hivltr.fsa [16], is indicated at the left of this first letter of each sequence displayed. When several sequences are re-grouped because they are identical (for instance 6 HIV-2-A sequences), the lowest and the highest coordinates are indicated. The sequences are often written in several lines to show similarities between sequences and parts of sequences.

Figure 5 shows the sequences re-written through the *N*-local decoding method definition (*N *= 11). Each letter followed by a number identifies an *N*-class (# indicates a number with more than one digit). Identical colours allow easy identification of repeated identifiers in the same column, and thus facilitate finding similar segments. Two kinds of similarity blocks have been identified in these 43 sequences. One exists in the NF*K*B binding sites, and the other is in NF*K*B flanking sequences.

(A) Similarity blocks in the NF*K*B binding sites reveal different duplication events of this site in various HIV clades. Each HIV-1 group M subtype has two NF*K*B site copies, with the exception of subtype C which exhibits an extra ≥ 10-letter long fragment (GGG(g)CgTTCCA) with 9 letters (here upper case letters) matching this NF*K*B site. Both HIV-1 group N sequences have two NF*K*B binding sites and one or two incomplete copy of such site (GGGACTTT), and these N-group sequences are more similar to SIV-CPZ and HIV-1-M than to other HIV clades. All HIV-1 group O sequences display two copies of a sequence fragment of 30 letters long (these two copies are written in two lines to show their similarity in Figure 5). Each fragment includes a NF*K*B binding site, a segment G_2_A_3_C_2_A_4 _similar to that in HIV-2 subtype B, and a segment C_3_T_#_G_#_C_5 _similar to SIV-CPZ-GAB. The differences in the similarity blocks in NF*K*B binding sites in HIV clades may indicate an important role the NF*K*B binding site played in different independent introductions of SIV-CPZ from chimpanzees into the human populations to establish HIV-1 M, N, O groups.

(B) Similarity blocks in NF*K*B site flanking sequences may also provide helpful information in tracking evolutionary relationships and distances between HIV clades. One example is the pattern G_#_C_5_A_1 _before the NF*K*B site, that only exists in HIV-2 and SIV-SMM sequences. Possibly this well-preserved G_#_C_5_A_1 _participated actively in the cross-transmission from SMM to human to establish the HIV-2. The other example is A_2 _A_# _G_# _G_# _G_# _pattern (in blue) at the 3' end of HIV-2 subtype A flanking sequences. This pattern distinguishes HIV-2 subtype A from other HIV clades. This, again, may simply reflect different transmission influences in varied HIV clades.

## Discussion

The current HIV sequence analyses usually involve obtaining a gap-stripped multiple alignment to construct an unbiased phylogenetic tree. The alignment usually is first generated by HMMER ([20,21] and/or other alignment softwares, followed by a manual editing [22]. After deleting the ambiguously aligned positions from the alignment, the final alignment leaves about half the sequence length [13]. This is a time-consuming procedure, and, most importantly, the alignment quite often underestimates the sequence variabilities, especially those embedded in the ambiguously aligned positions.

In this paper, we introduced a tree building method without a sequence alignment requirement. This *N*-local-decoding method calculates sequence dissimilarity matrices, based on re-writing, and re-classifies input sequences. Our HIV/SIV subtyping results showed that the classifications produced by this method agree very well with those obtained by a combination of standard methods. Thus comparing biological sequences without alignments appears to be an alternative to better explore sequence relationships.

However, there exist some discrepancies between our *N*-local-decoding-method-calculated trees and those obtained from standard methods. The differences may simply suggest that the *N *parameter used in *N*-local-decoding-method needs to be better defined, or they may be a consequence of the fact that we include ambiguous regions that are often ignored by traditional methods.

The *N*-local-decoding-method is particularly useful in the analysis of sequence variety and in tracking the sequence evolutionary events when a good sequence alignment is not possible. Our *N*-local-decoding-method is meaningful from the evolutionary point of view. Its success in sequence subtyping relies on capturing (im)perfect repeats or conserved regions in sequences (similarity blocks that are either closely or remotely related, and the latter one is often undetected by traditional methods due to removal of ambiguous alignment regions). The similarity blocks include internal repeats in one sequence or conserved regions among sequences, and these blocks are not necessarily to appear in the same order in the original sequences. In our method, the inversion could be detected by including the reverse complementary sequences in the sequence set.

This method is also practically applicable in terms of computing time and convenience to use. All the calculations in this paper have been done within a few seconds on a regular PC. The quality of the algorithm [8] is responsible for this speed. This algorithm has a complexity linear in the total length of the set.

The *N *value, the only parameter used in this method, could be set empirically according to the *N *values listed in the Results section.

Our method thus provides an alternative way of constructing sequence trees. It is helpful in tracking sequence information embedded in ambiguous alignment regions. In addition, the possibility of comparing sequences of varied lengths also suggests its direct use in detecting sequence recombinants. Finally, the similarity blocks found by this method could also be used as the anchor points for those similarity-block-based alignment programs to refine the quality of the alignment.

## Conclusion

The *N*-local-decoding method has shown its satisfactory applications in HIV/SIV subtyping. The deduced classifications are in good agreement with our best taxonomic knowledge, even for non-coding LTR regions that are not tractable by regular alignment methods due to frequent duplications/insertions/deletions. However, its application is not limited in HIV/SIV subtyping. In general, the advantages of this method are:

1) It generates sequence dissimilarity matrices without requiring sequence alignments.

2) It has only one user-specified parameter (*N: *the order of the site neighborhoods).

3) It is fast.

Thus the *N*-local-decoding method provides a promising approach to the rapid construction of trees without a time-consuming step of aligning sequences.

## Methods

### Sequences

Our test sequences include 59 HIV (Human Immunodeficiency Virus) and 7 SIV (Simian Immunodeficiency Virus) non-recombinant full length genome sequences and 4 incomplete non-recombinant HIV-2 sequences. Recombinants and non-recombinants are very well defined sequences [23]. These 66 complete genome sequences ranging in length from 8555 to 11443 nucleotides cover the following clades:

(1) HIV-1 group M (subtypes A-D, F-H, J, K; subtype A is further split into A1 and A2, and subtype F is divided into F1 and F2),

(2) HIV-1 group N,

(3) HIV-1 group O,

(4) HIV-2 (subtypes A, B, G),

(5) SIV-CPZ, and SIV-SMM

Four incomplete genome sequences are obtained from HIV-2 subtypes C, D, E, F, with lengths varying from 771 to 781 nucleotides, that cover about half of *gag *region. Thus those well-characterized 70 sequences, representing different HIV/SIV groups, sub-groups, subtypes, and sub-subtypes, are good samples of the genetic variation of the HIV/SIV world. These sequences can be retrieved from the Los Alamos HIV sequence database [24]. Their accession numbers are given in Figure 1. The sequence files are also available from [16].

### Algorithm

The sequence data described above were analysed using the method introduced in [7,8], which we call «local decoding of order *N*» of a sequence. Basically, the aim of the method is to find local similarities between (and within) the sequences of an input set. The input consists of a set of non-aligned sequences or a single sequence. In the input, any site of a sequence is occupied by a letter (typically a base or an amino acid). In the output, every site is occupied by an identifier that carries information about the letter and its neighborhood. The details of this method are described below.

A sequence *S *consists of letters *S*_1_...*S*_i_... with i running from 1 to |*S*|, the length of the sequence. Let *N *be a chosen integer parameter, which determines the size of neighborhoods to be considered. Consider the set of all overlapping words of length *N *(step 1) in the sequence *S*. Given a site (i.e. an integer i with 1 ≤ i ≤ |*S*|), consider the set of words that cover this site. Two sites are said to be «directly related» if they appear at the same position in two occurrences of the same word of length *N *(see example below). It is easy to see that two directly related sites have to be occupied by the same letter. Note that two sites may not be directly related but each of them may be directly related to a third site. More generally, two sites are said to be «related» (not necessarily directly) if they are linked by a chain of direct relations. In mathematical terminology, we consider the transitive closure of the direct relation. One can show that any two (even not directly) related sites are also occupied by the same letter. Finally, one obtains a decomposition of the set of all sites into disjoint classes (so called «*N*-classes»), each class being defined to be a maximal subset of sites that are pairwise related. This decomposition is always finer than (or the same as) the decomposition of sites into classes of sites occupied by a same letter. In this sense, local decodings of sequences are more informative. This method can be extended to a set of sequences. A site is then labelled by a pair (sequence name, position).

Using this method, we have re-written a set of HIV sequences in a larger alphabet. A «letter» of the new alphabet at a given site of the sequences, can be for instance «A215» where «A» is the letter in the input sequence at this site, and where A215 is the *N*-class identifier used to distinguish «related sites» (i.e. all sites carrying the symbol «A215») from non-related sites. This re-writing depends only on the choice of the integer parameter *N*. In addition, the re-writing of a set of sequences depends also on this sequence set itself, and could be changed if sequences are added or removed. In other words, «relatedness» is not an intrinsic property of sites.

### An example

**Figure 6:**
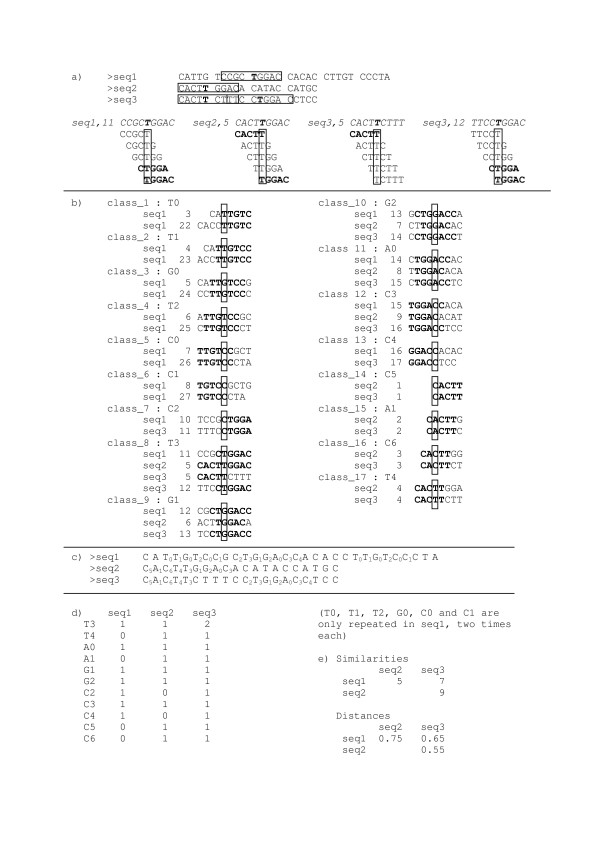
**The «local decoding of order *N*» computing strategy**. **Figure 6a (top)**: Four (*N *= 5)-related sites (containing the letter T) are taken from three input nucleotide sequences seq1, seq2 and seq3. Each of the four boxed sectors (2*N *- 1 = 9 letters in length) has T at its center (in bold face type) and is identified by the sequence where it is situated and the position of T in this sequence (that is seq1,11, seq2,5, seq3,5 and seq3,12, see Figure 6a bottom). **Figure 6a (bottom)**: each 9-letters-long segment (identified by the corresponding site containing a T in bold face type) is displayed with the set of corresponding overlapping (step 1) words of length *N *= 5 underneath the corresponding site (boxed). The four sites are 5-related; seq1,11, seq2,5 and seq3,12, are directly 5-related by TGGAC (in bold face type) at the position 1; seq1,11 and seq3,12 are also directly 5-related by CTGGA at the position 2; seq3,5 is directly 5-related with only seq2,5 by CACTT at the position 5, so that it is connected by seq2,5 with the other two sites. **Figure 6b**: the symbols that identify each class containing at least two sites, are shown together with the segments covered by the overlapping 5-words that lie over the letter (boxed). **Figure 6c**: the re-written sequences generated by the program. The identifiers corresponding to classes containing only one site are only represented by their corresponding letter in the input sequence; in fact, they cannot contribute to calculating the similarities between pairwise compared re-written sequences. **Figure 6d**: the double-entry table for constructing a pairwise distance matrix between the three sequences (re-written in **figure 6c**). Each class identifier with at least two sites is indicated in the corresponding row. For each row and for each of the three sequences that label the three columns, the table gives the number of sites of this *N*-class that appear in the sequence. **Figure 6e**: similarity matrix and the corresponding normalized dissimilarity matrix (see text) for the three sequences.

Figure 6a shows three sequences, named seq1, seq2, seq3, of length 30 nt, 20 nt, 20 nt, respectively. We choose *N *= 5. Consider the site at position 11 (letter T) in seq1. We say it is related with the site at position 5 in seq2, and with sites at positions 5 and 12 in seq3. Indeed, the site (seq1,11) is at position 1 in the word *TGGAC*. The same is true for (seq2,5) and (seq3,12). According to our «local decoding of order *N*» definition, these three sites are directly related to each other. The fourth site (seq3,5) is directly related to (seq2,5), because (seq3,5) and (seq2,5) are both at position 5 in the word *CACTT*. Consequently, the four sites (occupied by the letter T) are all pairwise related. They correspond to class_8 in Figure 6b and are identified by T3 in the output sequences (Fig. 6c).

Figure 6b shows all classes of sites containing at least two members, and Figure 6c shows the output of the program based on the algorithm described in [8]. Each site in the original sequences is replaced by the identifier of the *N*-class to which the site belongs. As mentioned above, the identifier consists of the letter occupying the site in the original sequence, followed by a number.

### Similarity and dissimilarity between sequences

Among many possibilities of defining a similarity between sequences, we choose the one described in [8]. In the program output (Fig. 6c), every site in the compared sequences gets occupied by an identifier. We need an extra notation here and denote by |w|_x _the number of occurrences of an identifier x in any sequence (w). Let us first introduce a similarity score between two sequences u and v. Each identifier x could occur in any alignment (alignments are not used by our method) of u and v in at most min(|u|_x_,|v|_x_), that is the smallest number of occurrences of x in the two sequences u and v. Adding up this number over all identifiers gives an upper bound of the maximum number of possible matches between u and v (Fig. 6d). Finally, we normalize the obtained similarity by dividing it by the length of the shorter sequence (this makes it possible to subtype short sequence parts together with complete sequences, see **Results **first section). It is then straightforward to obtain a dissimilarity score, that is 1 minus the normalized similarity (Fig. 6e). Figure 6d–e outlines the method for calculating the dissimilarity matrix between the three compared sequences re-written in Figure 6c, by using the *N*-local decoding method. For instance, when comparing seq1 and seq2, the sum of all the min(|u|_x_,|v|_x_) equals 5, so that the corresponding dissimilarity is 1 - (5/20) = 0.75 (Fig. 6e). Here, the identifiers that correspond to classes containing only one site have been omitted, since they cannot contribute to the similarity (Fig. 6c).

### Computational programs used in this study

A program implementing the *N*-local decoding method was used for constructing HIV/SIV trees. It reads a set of FASTA input sequences, and generates a tree based on the dissimilarity matrix described above. This program is available from [16]. A more recent program [8] written in C, and the supplementary material are available upon request from GD (address in the title page).

Other programs used in this paper are: CLUSTAL-W (version 1.8 June 1999) [18], DIALIGN-2.2.1 [19] and PHYLIP [25]. Some of these programs have been used to compute the alignment-based dissimilarity matrices from which the trees have been constructed for Figure 3 (CLUSTAL-W) and for Figure 4 (DIALIGN-2.2.1).

### Computing bootstrap values over trees

To assess the reliability of the trees computed from the dissimilarity matrices, we use a bootstrapping procedure similar to the one proposed in [26] which proceeds by resampling the columns of an alignment. Though we do not deal with an alignment but just a set of local decoded sequences, a very similar procedure can be applied here. Practically, from the local decoded set of sequences, we resample a given number of bootstrap replicates. These replicates are the sets in which each sequence of the initial local decoded set is replaced by a sequence of the same length. This sequence is generated by concatenating randomly selected sites in the sequence of the initial set (some positions can be selected more than once, others never selected). For all the resampled sets of sequences we compute dissimilarities and associated neighbor-joining trees. Those trees are finally combined using the «consense» program of PHYLIP [25] to give a final tree and related bootstrap values.

## Authors' contributions

The local decoding algorithm and the program code were developed by GD. LD and MP calculated the dissimilarity matrix and implemented the user interface. IL initiated and led the application to HIV/SIV on the basis of his previous work on the subject. IL wrote the manuscript together with AG, CD and MZ. AG and CD contributed mainly to the section about the method, while MZ played an important role in the presentation and discussions of biological aspects of the paper. All authors read and approved the final manuscript.
